# A computational model of altered gait patterns in parkinson's disease patients negotiating narrow doorways

**DOI:** 10.3389/fncom.2013.00190

**Published:** 2014-01-09

**Authors:** Vignesh Muralidharan, Pragathi P. Balasubramani, V. Srinivasa Chakravarthy, Simon J. G. Lewis, Ahmed A. Moustafa

**Affiliations:** ^1^Department of Biotechnology, Indian Institute of Technology MadrasChennai, India; ^2^Parkinson's Disease Research Clinic, Brain and Mind Research Institute, The University of SydneySydney, NSW, Australia; ^3^Marcs Institute for Brain and Behaviour and School of Social Sciences and Psychology, University of Western SydneySydney, NSW, Australia

**Keywords:** gait, freezing of gait, doorway, basal ganglia, reinforcement learning

## Abstract

We present a computational model of altered gait velocity patterns in Parkinson's Disease (PD) patients. PD gait is characterized by short shuffling steps, reduced walking speed, increased double support time and sometimes increased cadence. The most debilitating symptom of PD gait is the context dependent cessation in gait known as freezing of gait (FOG). Cowie et al. (2010) and Almeida and Lebold (2010) investigated FOG as the changes in velocity profiles of PD gait, as patients walked through a doorway with variable width. The former reported a sharp dip in velocity, a short distance from the doorway that was greater for narrower doorways. They compared the gait performance in PD freezers at ON and OFF dopaminergic medication. In keeping with this finding, the latter also reported the same for ON medicated PD freezers and non-freezers. In the current study, we sought to simulate these gait changes using a computational model of Basal Ganglia based on Reinforcement Learning, coupled with a spinal rhythm mimicking central pattern generator (CPG) model. In the model, a simulated agent was trained to learn a value profile over a corridor leading to the doorway by repeatedly attempting to pass through the doorway. Temporal difference error in value, associated with dopamine signal, was appropriately constrained in order to reflect the dopamine-deficient conditions of PD. Simulated gait under PD conditions exhibited a sharp dip in velocity close to the doorway, with PD OFF freezers showing the largest decrease in velocity compared to PD ON freezers and controls. PD ON and PD OFF freezers both showed sensitivity to the doorway width, with narrow door producing the least velocity/ stride length. Step length variations were also captured with PD freezers producing smaller steps and larger step-variability than PD non-freezers and controls. In addition this model is the first to explain the non-dopamine dependence for FOG giving rise to several other possibilities for its etiology.

## Introduction

Altered gait behavior is a motor impairment observed in patients with Parkinson's disease (PD), a neurodegenerative disorder that involves a loss of dopaminergic neurons in the brain. PD gait is characterized by the following features: (1) Reduced stride length, reduced walking speed, increased cadence and increased double support duration (Morris et al., 1998); (2) Exhibits flat foot strike, and in rare conditions the “toe to heel strike” gait pattern is also observed (Hughes et al., 1990); (3) Intra-individual variability in foot strike patterns is lower in PD patients than in control subjects (Kimmeskamp and Hennig, 2001); (4) Vertical ground reaction force (VGRF) representing the normal force exerted on the foot during gait, has two peaks in controls—one when the foot hits the ground, and the other when it lifts off again. In early stages of PD, the two peaks in VGRF are present but with lower intensity compared to controls. In advanced PD, where the patients walk with narrow shuffling steps, the two peaks in VGRF merge into one (Koozekanani et al., 1987); (5) Postural instability is a common feature in late stage PD. Postural sway is also reduced probably due to reduced flexibility in adjusting one's bodily responses to changing posture (Morris et al., 2000). Abnormal postural sway in PD might also be due to stiff joints. The degree of gait variability as seen by any of the above mentioned features, is correlated with gait severity in PD patients (Hausdorff et al., 1998).

In addition to the aforementioned features, a more debilitating and dramatic feature of PD gait is known as Freezing of Gait (FOG). It is characterized by frequent falls (Latt et al., 2009), and is an episodic phenomenon of cessation of gait triggered by certain environmental contexts like narrow passages or crowded places (Almeida and Lebold, 2010; Cowie et al., 2010). PD gait features like reduced stride length and reduced walking speed appear to be gradually aggravated under certain environmental conditions, culminating in a motor block, or a freezing episode (Chee et al., 2009). Some cases of PD patients (PD-freezers) exhibit freezing in specific contexts such as facing transverse lines on a road crossing or narrow doorways (Hughes et al., 1990; Morris et al., 1998), while the same transverse lines on a treadmill alleviates freezing symptoms (Azulay et al., 1999). This shows the importance of the higher level cortical control over the rhythm generating spinal control in gait and FOG, since the visual feedback can affect gait only through the cortical route.

**Figure 1:**
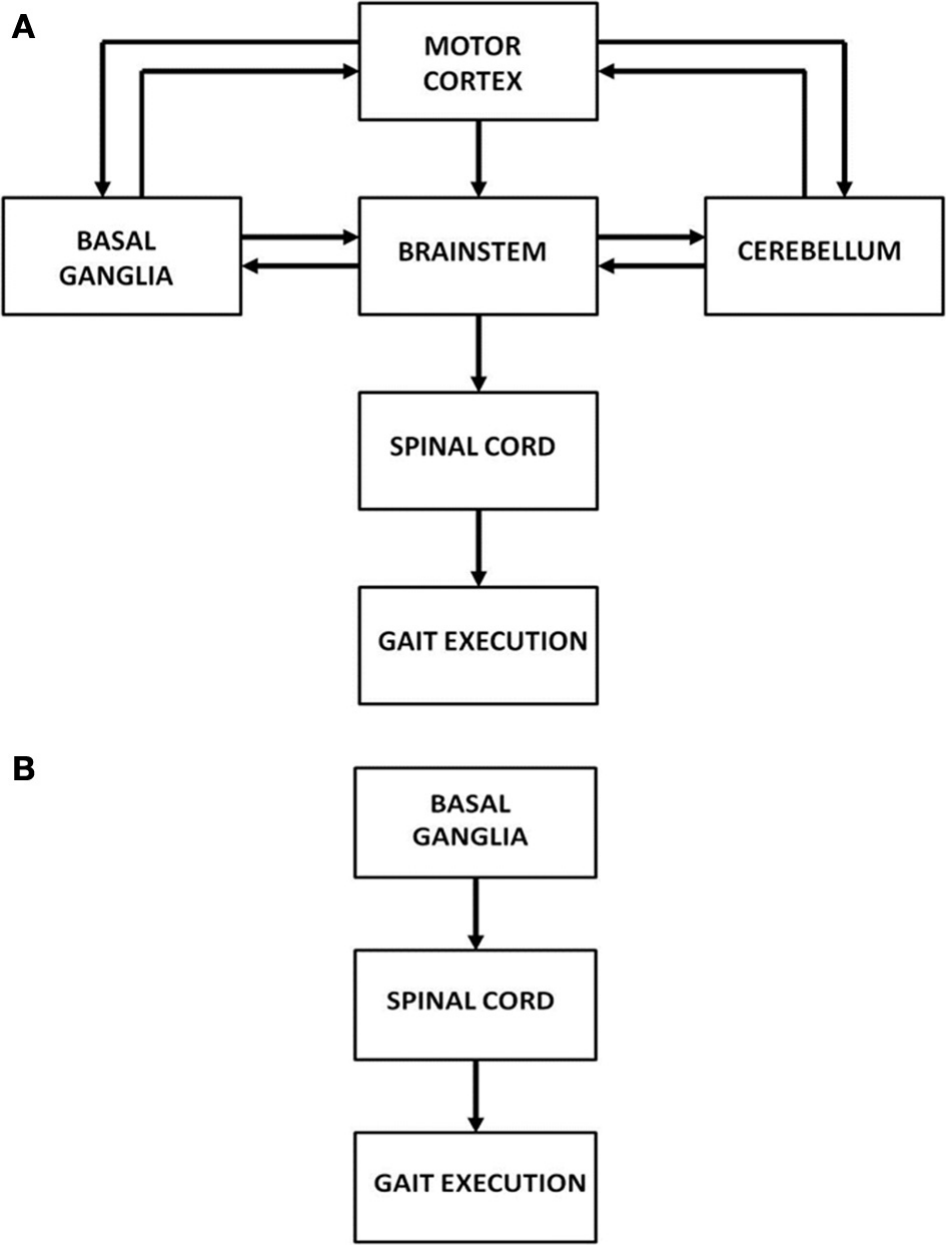
**(A)** Architecture showing the hierarchy of control on gait execution; **(B)** Model architecture considered in our study to understand FOG.

Human motor function has three levels of control: cortical, subcortical and spinal. Specifically gait is controlled by a complex network of brain areas spanning all the three levels: the neocortex (Sahyoun et al., 2004); subcortical areas including the basal ganglia (BG), vestibular system, cerebellum; and the spinal cord (Middleton and Strick, 2000; Lemon, 2008; Takakusaki et al., 2008). Motor commands arising from the brain's gait control centers are strongly influenced by sensory feedback via visual, proprioceptive and other sensory channels (Sahyoun et al., 2004). At the level of spinal cord each limb is thought to be controlled by a network of unit burst generators called Central Pattern Generators (CPGs) (Ijspeert, 2008). This network of CPGs, which acts under the top-down control from higher cortical motor areas, and the proprioceptive and visual feedback, is thought to be the ultimate driver of human gait. The broad picture of the neural substrates involved in gait control is shown in Figure 1A. However, since the focus of the present study is PD gait, we limit ourselves to a smaller architecture that highlights the role of BG (Figure 1B). A more detailed description and justification of the model architecture is presented in The Model.

Motor and other forms of impairment observed in PD are primarily linked to dopamine deficiency caused by cell loss in the Substantia Nigra pars compacta (SNc), a small but important nucleus in BG (Kish et al., 1988). The BG is a group of subcortical nuclei performing vital roles of action selection, action gating, motor preparation, among others (Chakravarthy et al., 2010). The striatum is the major input port of BG affected by the activity of the cortex and the limbic regions. This gets connected directly to the output port (Globus Pallidus interna / Substantia Nigra pars reticulata) via the direct pathway (DP), or through Sub-thalamic Nuclei—Globus Pallidus externa network via the indirect pathway (IP). The output nuclei project onwards to cortical targets like prefrontal, premotor and the motor cortices via the thalamus (Chakravarthy et al., 2010).

The idea that mesencephalic dopamine signal is linked to environmental rewards (Houk et al., 1995; Schultz et al., 1997) opened doors to the application of concepts from reinforcement learning (RL) to model BG (Joel et al., 2002; Frank, 2005; Chakravarthy et al., 2010). The basic tenet of RL is that stimulus-response pairs that are rewarding are reinforced and those that are punitive are attenuated. The mapping between stimuli and responses would have been an easier problem, but for the fact that often reward comes, not immediately after an action is performed, but after a delay. In some cases, reward and punishment feedback arrives after a long series of actions. It remains then to allocate credit to past actions and determine which actions have contributed to reward and to what action, a problem otherwise known as temporal credit assignment problem. Since reward comes after a delay, for the simulated object (referred to here as agent) to select the correct action at any given instant, RL theory offers a surrogate to reward known as value function. The value function is defined as the total expected future reward, with appropriate discounting of future. The RL component known as the 'Critic' computes value after repeatedly sampling the action space and receiving rewards/punishments. Another key RL component known as the 'Actor' uses the value information provided by the critic to select correct or potentially rewarding actions. Many computational models have been directed toward mapping RL concepts onto the functional anatomy of BG (Joel et al., 2002).

According to the classical depictions of functional anatomy of BG, the DP facilitates movement, and is hence dubbed the GO pathway, while the IP inhibits movement and hence known as the NOGO pathway (Albin et al., 1989; Frank, 2005). Signal transmission between DP and IP is thought to be switched by striatal dopamine: higher (lower) levels of striatal dopamine activate the GO (NOGO) pathway. In earlier work, we proposed that the classical GO/NOGO picture of BG function needs to be expanded, suggesting insertion of a third “EXPLORE” regime between GO and NOGO (Sridharan et al., 2006; Chakravarthy et al., 2010; Magdoom et al., 2011; Kalva et al., 2012). This EXPLORE regime drives the stochastic exploration of action space, which is essential for RL to work in complex environments. In the present study we use this expanded GO/EXPLORE/NOGO (GEN) understanding of BG functioning, to model PD gait.

Two experimental studies, that investigate the gait pattern of PD patients as they approach a doorway, are simulated in the present study (Almeida and Lebold, 2010; Cowie et al., 2010). The study of Cowie et al. (2010) shows a sharp dip in velocity as the PD patient approaches the doorway, a dip that becomes sharper in the case of narrower doorways (Cowie et al., 2010); this effect was more pronounced in PD patients (ON and OFF freezers) than in healthy controls. Almeida and Lebold (2010) consider a similar setup but compare the gait patterns of PD freezers with non-freezers in terms of step lengths and its variability (Almeida and Lebold, 2010). The proposed BG model accounts for the above mentioned velocity profiles and gait features (stride / step lengths) of PD patients from these two experimental studies.

We model them at two stages of control: (1) the higher level of control representing the cortico-basal-ganglia system, and (2) the spinal level CPGs that translate the higher level gait commands such as velocity into gait rhythm (Figure 1B). The BG model is essentially simulated using the Actor-Critic architecture, with the difference that the Actor is modeled by the GEN model (Sridharan et al., 2006; Chakravarthy et al., 2010; Magdoom et al., 2011; Kalva et al., 2012). The spinal CPGs are modeled by networks of hopf oscillators (Righetti and Ijspeert, 2006). The model is used to simulate the results of two PD gait studies (Almeida and Lebold, 2010; Cowie et al., 2010).

The paper is outlined as follows: the Model section describes the modeling components and equations. The Result section explains the experimental setup, the model implementation and the simulation results. Velocity profiles of control subjects and PD patients (ON/OFF, freezers / non-freezers) as they negotiate a doorway are simulated and compared with experimental results. Section Discussion finally discusses the results obtained, model limitations, predictions and future work.

## The model

The proposed model simulates the approach of a subject to a doorway and computes the velocity profile along the track leading to the doorway. The agent repeatedly approaches a doorway, walking along a short track. The agent aims at passing through the doorway without bumping into the sides of the doorway. Due to the well-known tradeoff between accuracy and speed in motor function (Mackay, 1982; Bradshaw and Sparrow, 2000; Duarte and Latash, 2007), rapid approaches to the doorway are more likely to result in a collision. Therefore, in our model, the agent learns to reduce its speed in the vicinity of the doorway, which it does using RL mechanisms.

**Figure 2:**
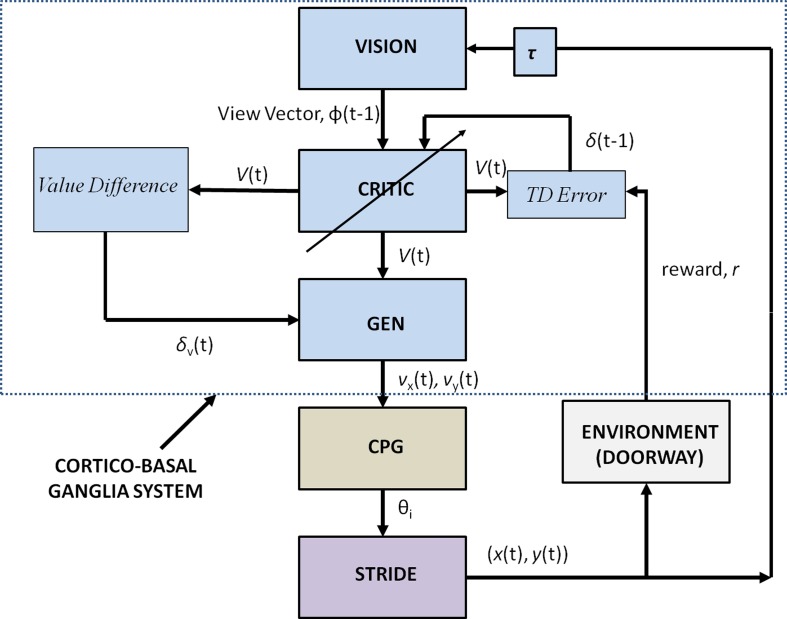
**Block diagram detailing the Cortico-Basal Ganglia system and the Central Pattern Generator module used in our study**. The arrow on the Critic represents the module training. The figure also projects the Cortico-BG system, CPG, and locomotor apparatus in the shades of blue, brown, and violet respectively.

Figure 2 shows the block diagram of the proposed model, which mainly consists of three components—the Cortico-BG system, CPG, and locomotor apparatus. The Cortico-BG system, shown inside the dashed box (Figure 2), takes a representation of the view of the doorway, the “view vector,” from the position, *X*, of the agent. It is obtained from the cortical module: VISION. The block denoting τ denotes the time delay in the passage. The BG [consisting of the CRITIC, ACTOR (GEN), VALUE DIFFERENCE, and the TD ERROR modules] uses the view vector and updates the agent's velocity (*v_x_* and *v_y_*). This velocity information from the higher command centers is sent to the CPG module, which translates the velocity into joint angles (θ). The subsequent block labeled STRIDE uses the joint angle information and orientation (*v_x_* and *v_y_*) and computes the next position. The ENVIROMENT (doorway) module checks if the new position results in a collision of the agent with the doorway. A positive reward, *r*, is delivered if there is no collision, and a punishment (negative *r*) in case of collision. The BG uses the view vector and reward information to compute value, thereby completing the cycle.

We now describe individual model components in detail.

### The cortico-basal ganglia system: vision

**Figure 3:**
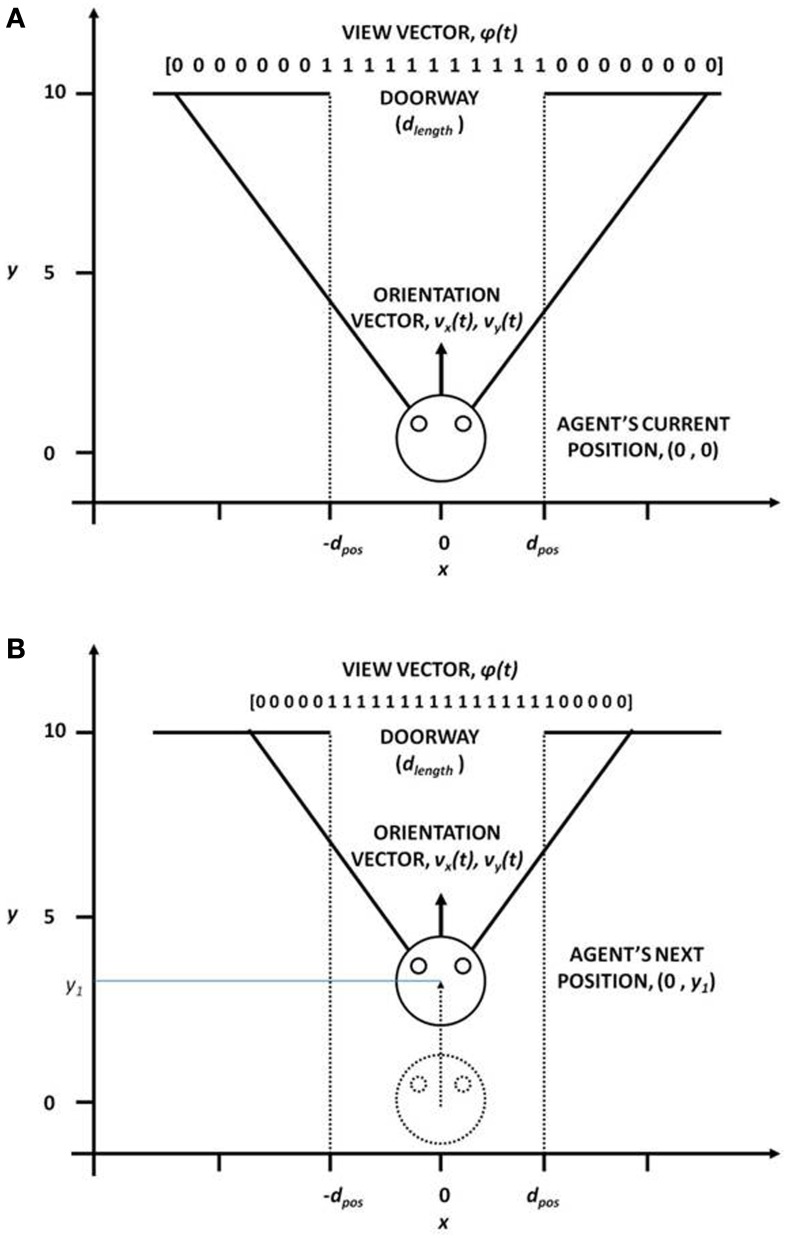
**View vector associated with different (*x, y*) positions and orientation vector for **(A)** far from the doorway and **(B)** near the doorway, for the fixed door size (*d*_length_)**.

This module computes the state of the agent, the “view vector,” ϕ, which codes the view of the doorway from the position [(*x, y*) or *X*] of the agent (Figure 3). The calculations are given by the Equations (1–5).

In our study, the field of vision (FOV) of the agent is fixed at 120°. The FOV is divided into small sectors, denoting the size of the view vector. In our case, the view vector is a 1x 50 array and therefore FOV is spilt into 50 sectors (Figure 3). The position of the agent (*x, y*) is the viewing point and the orientation vectors *v_x_* and *v_y_* form the view direction of the agent from which it can see 60° to the left and 60° to the right. Considering *R*_o_ as the orientation vector (2 × 1) represented by *v_x_* and *v_y_* and the angle subtended by each *i*th sector with respect to *R*_o_as Θ*^sec^_i_*, the orientation vectors of each of other 49 sectors is given by

(1) $$R_{i}^{\text{sec}}=O_{\text{mat}}.R_{\text{o}}$$

where *O*_mat_ is the orientation matrix (2 × 2) given by

(2) $$O_{\text{mat}}=\left[\cos\left(\Theta_{i}^{\text{sec}}\right),\sin\left(\Theta_{i}^{\text{sec}}\right);-\sin\left(\Theta_{i}^{\text{sec}}\right),\cos\left(\Theta_{i}^{\text{sec}}\right)\right]$$

The slope *m_i_*(Equation 3) of each of the *R^sec^_i_* is calculated with respect to the agent's current position (*x, y*).

(3) $$m_{i}=\left[\left(y+R_{i}^{y}\right)-y\right]/\left[\left(x+R_{i}^{x}\right)-x\right]$$

In order to identify if a given sector's orientation hits the door or a wall assuming the y coordinate of the door is *y*^door^_*i*_, the x-coordinate (*x*^door^_*i*_) of each of the orientation vectors is calculated at *y*^door^_*i*_ as in Equation 4.

(4) $$x_{i}^{\text{door}}=\left(y_{i}^{\text{door}}-y\right)/m_{i}+x$$

Using the *x*^door^_*i*_ coordinates of all the views, the view vector is given as Equation 5.

(5) $$\begin{array}{l} \text{if}{\left(x_{i}^{\text{door}}\geq -d_{{\text{pos}}_{x}}\right)}^{\wedge }(x_{i}^{door}\leq d_{{\text{pos}}_{x}})\\ \;\phi_{i}\left(t\right)=1\\ \text{else}\\ \;\phi_{i}\left(t\right)=0 \end{array}$$

Therefore, the agent viewing the doorway from a given position (*X*), would see more or less number of 1 s in its visual field, depending on its orientation, distance to the doorway and the width of the doorway (*d*_length_) (Figure 3). The view vector is thus ideally suited to be used as the state of the agent.

### The basal ganglia module

The BG module is essentially simulated using the Actor-Critic architecture (Joel et al., 2002) but with important deviations from classical RL (Sutton and Barto, 1998) regarding the formulation of the Actor.

#### Critic

The Critic computes the value “*V*” for the view vector [ϕ(*t*)]. It is defined as an estimation of the predicted reward at any time, *t*, for that state ϕ(*t*). The value function is denoted by Equation 6.

(6) $$V(t)=E(r_{t+1}+\gamma r_{t+2}+\gamma^{2}r_{t+3}+\cdots )$$

Here, r_*t*_ is the reward *r* obtained at time, *t*.

In our study, we approximated *V*(*t*) as in Equation 7.

(7) $$V(t)=\tanh\left[\sum W_{\text{i}}(t)\phi_{\text{i}}(t)\right]$$

The update equation for the above approximation (having weight vector, *W*) is given by eqn. 8.

(8) ΔW=ηδϕ(t)

Here, “δ(*t*)” denotes the TEMPORAL DIFFERENCE (TD) error in value function, that is correlated to dopamine signaling (Schultz, 2010). It is given by Equation 9 in which γ is the discount factor.

(9) δ=r(t)+γV(t)−V(t−1)

#### GO/EXPLORE/NOGO or GEN

The policy (Actor) used here is known as the GO/EXPLORE/NOGO or GEN policy, the neurobiological origins of which were described in earlier work (Sridharan et al., 2006; Magdoom et al., 2011; Kalva et al., 2012). GEN essentially represents an approach to action selection, by performing a stochastic hill-climbing over the value function. In the doorway problem that is presently studied, reward, *r*, is obtained at the doorway when the agent passes through the doorway without collision. Thus the value profile is expected to have a maximum at the doorway. Therefore, value gradient can be used to approach the doorway securely without colliding with the sides of the doorway.

A quantity known as *VALUE DIFFERENCE* (Equation 10), δ_*V*_, which is the gradient of the value,

(10) $$\delta_{V}=V(t)-V(t-1)$$

plays an important role in the process of hill-climbing over the value profile.

Note the resemblance between the Value Difference in eqn. 10 and the TD error (Equation 9). It may be observed that, δ = δ_*V*_, when γ = 1 and when the agent is not at the goal state (*r* = 0). We assume that both δ and δ_*V*_ represent dopamine signals but perform distinct roles: while δ is used for training the value function as in the case of typical Actor-Critic models of BG, we assume that δ_*V*_ is used for switching between DP and IP, which is thought to be a function of striatal dopamine (Humphries and Prescott, 2010; Amemori et al., 2011). δ_*V*_ can be used to hill-climb over value function using the following rules,

(11) $$\begin{array}{l} \text{if}(\delta_{V}>D_{\text{hi}})\\ \;\begin{array}{ccc} \Delta X(t)=+\Delta X(t-1) & -\text{"Go"} & \;(a) \end{array}\\ \text{elseif}(\delta_{V}>D_{\text{lo}}\wedge \delta_{V}\leq D_{\text{hi}})\\ \begin{array}{ccc} \;\Delta X(t)=\chi & \;-\text{"Explore"} & \left(b\right) \end{array}\\ \text{else }(\delta_{V}\leq D_{\text{lo}})\\ \;\begin{array}{ccc} \Delta X(t)=-\Delta X(t-1) & \text{ ​}-\text{"NoGo"} & \;(c) \end{array} \end{array}$$

where *X* = (*x, y*) denotes the position of the agent on the track; *D*_hi_is a positive threshold and *D*_lo_ is a negative threshold; χ is a uniform random variable. A similar rule for hill-climbing over value function was used earlier in Magdoom et al. (2011), which describes a model of Parkinsonian reaching movements.

The key difference in the classical RL implementation of Actor, wherein the action is typically modeled as an explicit function of the state ϕ, and the GEN policy, is that the action is computed by following the value gradient over the position space, *X*. Although value is a function of the view vector, ϕ(*t*), we perform the hill-climbing over the position space, *X*, that is mapped onto the view vector uniquely.

The 3 discrete regimes—GO, EXPLORE and NOGO—of Equation 11 can be combined seamlessly into a single equation as follows (Equation 12):

(12) $$\begin{array}{l} \Delta X(t)=A_{\text{G}}sig(\lambda_{\text{G}}\delta_{V})\Delta X(t-1)\\ \;+A_{\text{E}}\chi \exp(-\delta_{V}^{2}/\sigma_{\text{E}}^{2})\\ \;-A_{\text{N}}sig(\lambda_{\text{N}}\delta_{V})\Delta X(t-1) \end{array}$$

where

(13) $$sig(x_{sig})=1/\left[1+\exp(-x_{sig})\right]$$

**Figure 4:**
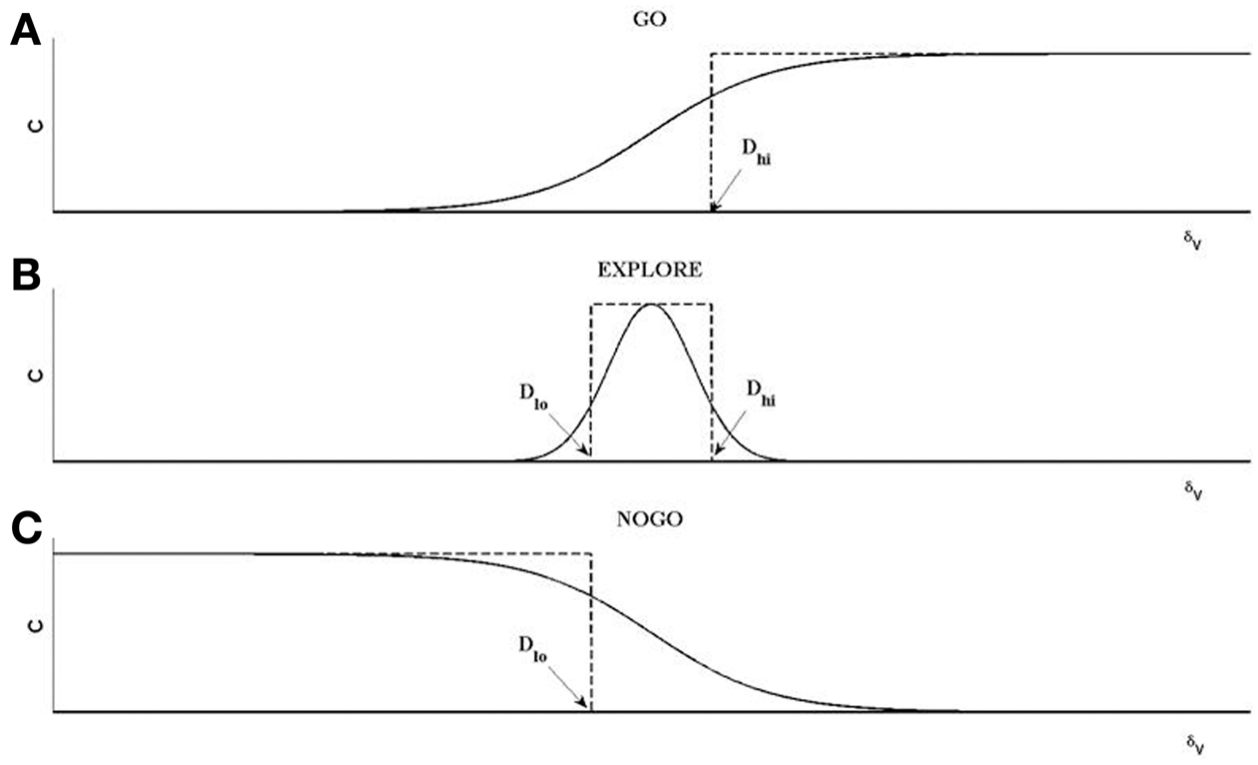
**An illustration of the operation of GO, EXPLORE, NOGO regimes**. Each of the regimes represent a map between *X*_(*t*−1)_ and *X*_(*t*)_ defined as *X*_(*t*)_ = χ*C_(δ*v*)_*X_(*t*−1)_. **(A)** For GO regime, for δ_*v*_ > *D*_hi_, *C* = 1; else *C* = 0; χ = 1. The resulting step-like profile is approximated by a sigmoid, shown as the first term on the RHS of Equation (12). **(B)** For NOGO regime, for δ_*v*_ < *D_lo_*, *C* = 1, else *C* = 0; χ = 1. The resulting inverted step profile is approximated by the sigmoid defined as the third term on RHS in Equation (12). **(C)** For the EXPLORE regime, for *D*_lo_ < δ_*v*_ < *D*_hi_, *C* = 1, else *C* = 0. This pulse-like profile of *C* is approximated by a Gaussian function of δ_*v*_ a; χ is a random number generated from a uniform distribution with range [−0.5 to 0.5] in this case.

The rationale behind Equations (11–13) (Sridharan et al., 2006; Chakravarthy et al., 2010; Magdoom et al., 2011; Kalva et al., 2012) may be described as follows. The “GO” regime, which occurs when δ_*V*_ > *D*_hi_, means that the previous position update, *X*(*t* − 1), had caused significant increase in the value (δ_*V*_). Therefore, according to Equation 11 above, Δ*X*(*t*) is in the same direction as Δ*X*(*t* − 1), which justifies the form of the first term in eqn. 12, that is a continuous version of rule Equation 11a, as shown in Figure 4. The GO regime is thought to be implemented by the DP, which is activated at higher levels of striatal dopamine, δ_*V*_. A low level of dopamine δ_*V*_ < *D*_lo_ implies that the previous position update had caused significant decrease in the value. Therefore, the position update is in the opposite direction to the previous update. This mechanism is thought to be implemented by the IP. This regime is denoted by the third logsig term with a negative slope (λ_N_) in the Equation 12, a continuous version of the rule of Equation 11c. Intermediate levels of dopamine, (*D*_hi_ < δ_*V*_ < *D*_lo_), implies that the previous change in value is not significant; therefore the subsequent position update occurs in a random (χ) direction (Equation 11b). The second term in Equation (12) is a continuous version of the rule of Equation 11b.

The parameters that define the GEN policy are *A*_G_, *A*_N_, *A*_E_, λ_G_, λ_N_ in Equation (12), the discount factor, γ in Equation (9), and width, σ, of the Gaussian term in Equation (12). The last parameter, σ, is known as the “exploration parameter” since it controls the extent of exploration by the GEN policy. The parameters that denote changes in dopamine corresponding to PD OFF and ON conditions are δ_lim_ and δ_med_ respectively. These parameters are trained using genetic algorithms (Appendix A) after imposing specific constraints related to various conditions (controls, PD OFF and PD ON) as described in the later sections.

Thus the GEN policy computes the update in position, Δ*X*. The position update is represented as velocity components (*v_x_* and *v_y_*) and passed onto the CPG module, which in turn computes the hip and knee angles θ, for the calculation of the next position.

### The CPG module

**Figure 5:**
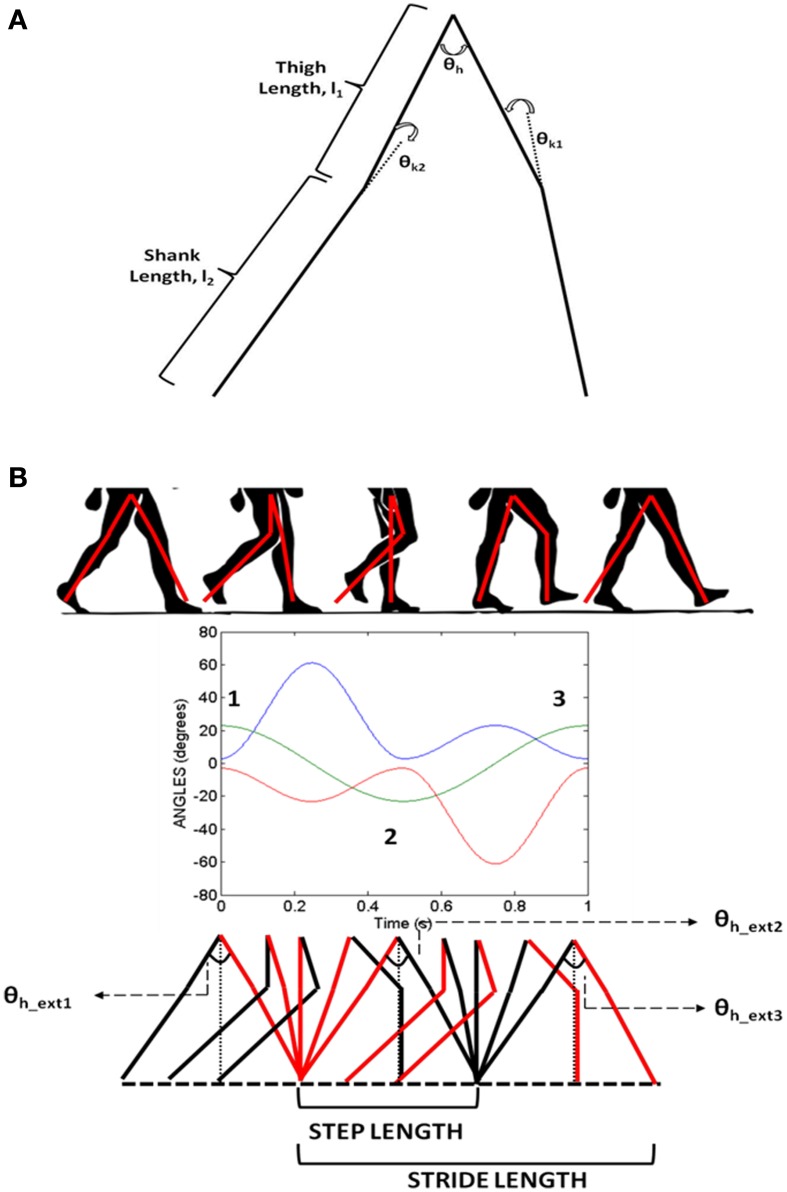
**(A)** The joint angle representation on the kinematic leg model with the thigh (l_1_) and shank (l_2_) links representing the joint angles θ_*h*_ (hip), θ_*k*1_, θ_*k*2_(two knees); **(B)** Variation of hip and knee angles with time and their inter-phase relationships. Extrema (θ_*h_ext*_) in the hip angle are denoted by numbers 1, 2, and 3.

CPGs are neural networks capable of producing coordinated rhythmic activity in the spinal cord for driving rhythmic movements like locomotion (Ijspeert, 2008). A network of coupled non-linear oscillators, modeled using adaptive hopf oscillators (Righetti and Ijspeert, 2006), is used here as a model of the CPG network. The model assumes that the CPG controls the angle profiles of hip and knee joints that directly reflects the motor output, producing the necessary activation and deactivation of muscles producing gait. It is a simple kinematic model of the leg, where the CPGs control the joint angles including those of the hip (θ_*h*_) and two knees (θ_*k*1_ and θ_*k*2_). The hip and knee joint angles are approximations of the human locomotion obtained by Fourier analysis (De Pina Filho and Dutra, 2009). Figure 5B shows the approximate profiles of hip and knee joints, modeled as truncated Fourier series (De Pina Filho and Dutra, 2009). It represents the training signals for the CPGs which are 500 steps in time for one gait cycle (T). Since our aim is to reproduce the rhythms, during training these are provided repeatedly to the network till it converges to produce such gait cycles with appropriate amplitude, frequency and phase relationship.

**Figure 6:**
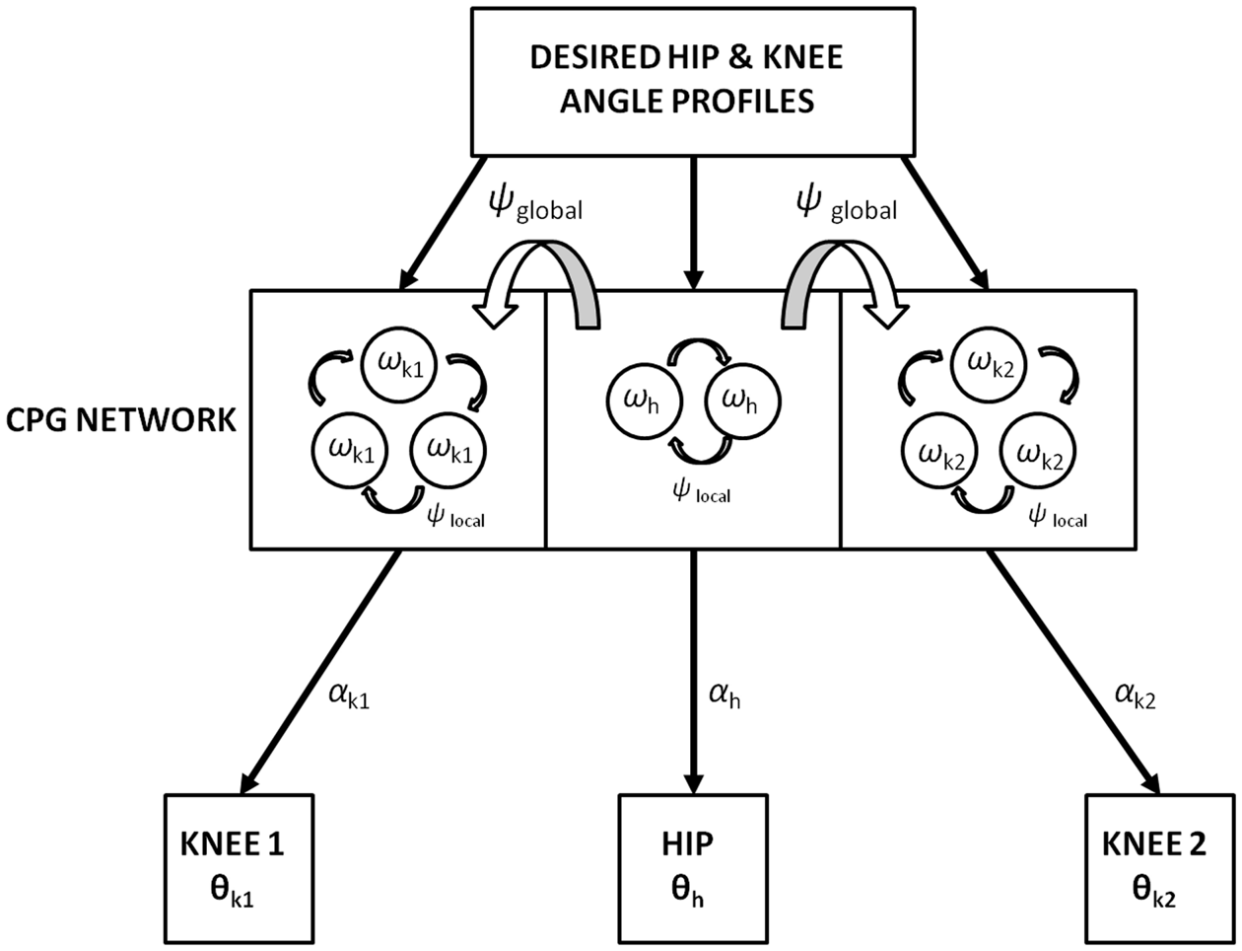
**Training of the CPG network with the desired hip (*h*) and knee (*k*1 and *k*2) angles (θ) represented in Figure 5B**. The number of hopf oscillators used to train the hip (ω_*h*_) and knee angles (ω_*k*1_ and ω _*k*2_) are 2 and 3 respectively. Phase difference within-CPGs is maintained by ψ_local_ while across-CPGs is maintained by ψ_global_. αs modulate the intrinsic CPG rhythm to output the learnt joint angles.

The motivation behind using the adaptive hopf network is to have a smooth control over the amplitude and frequency of the oscillators. Three pools are used to represent the CPG network (where s = 3 and *j* = 1: *s* represents the hip, knee1, and knee2 respectively). Each pool consists of optimal number of oscillators, two for the hip and three for each of the knees (where *N* = 1 or *N* = 2 and *i* = 0: *N*, respectively), that in total constitute the CPG network (Figure 6). The dynamics of the adaptive hopf oscillators are given by Equations 14–21, for the neurons (oscillators) in each pool *j*. Each variable is represented with the subscript *i*, *j* denoting the *i*th oscillator in the *j*th pool. The intrinsic variables *p*_*i, j*_and *q*_*i, j*_ of the oscillators are in Equations 14, 15 with $z_{i,\;j}=\sqrt{p_{i,\;j}^{2}+q_{i,\;j}^{2}}$. *F_j_*(*T*) is the error signal as described in Equation 18, where “*T*” denotes time steps needed to complete one gait cycle, which in our study is taken to be a vector of size [1 × 500]. It is weighted by a factor ϵ, and is given as feedback to the oscillators through Equation 14. In Equations 14, 15, μ controls the amplitude of oscillations, and ξ controls the speed of recovery of the system after perturbations.

(14) $$p_{i,j}=\xi (\mu -z_{i,j}^{2})p_{i,j}-\omega_{i,j}{\text{q}}_{i,j}+\varepsilon F_{j}(T)+\tau \sin(\theta_{i,j}^{IP}-\psi_{i,j})$$

(15) $$q_{i,j}=\xi (\mu -z_{i,j}^{2})q_{i,j}-\omega_{i,j}p_{i,j}$$

The adaptation of the oscillators to a specific frequency (ω*_i, j_*) and amplitude (α_*i, j*_)of an input signal is achieved by Equations 16 and 17 respectively. The learning rate η_*a*_for the update equation for α_*i, j*_ (Equation 17) is set at 0.08.

(16) $$\omega_{i,j}=-\varepsilon F_{j}(T)\frac{q_{i,j}}{z_{i,j}}$$

(17) $$\alpha_{i,j}=\eta_{a}p_{i,j}F_{j}(T)$$

*F*_*j*_(*T*) describes the error signal (Equation 18) that is defined as the difference between the teaching signal (*P*_teach_,*j*) and the learnt signal (*Q*_learned_,*j*) at a time instant. The teach signals (a single gait cycle) for the oscillators in a pool “*j*” represent the angle profile of any one of the joints (either the hip, knee1, or knee2) seen in Figure 5B and is vector of size (1 × 500) in time. Hence all the oscillators (*i* = 0: *N*) in a particular pool (*j*) receive the teach signal of the hip (if *j* = 1) or the knee 1 (if *j* = 2), knee 2 (if *j* = 3), respectively.

(18) $$F_{j}(T)\;=\;P_{teach,\;j}(T)-Q_{learned,j}(T)$$

*F*_*j*_(*T*) is provided to the oscillatory network only during the adaptation stage (learning) and grows smaller as the learning progresses till it eventually becomes zero (*P*_teach_,*j* = *Q*_learned_,*j*). The α_*i, j*_ and ω_*i, j*_ converge at this point, and the network still encodes the pattern even after the removal of *F*_*j*_(*T*). These variables can be represented as α^0^_*i, j*_ and ω^0^_*i, j*_ where the superscript “0” denotes the convergence to optimal values. The learnt signals which are joint angles, expressed here by θ_*h*_, θ_*k*1_and θ_*k*2_, are the output of each oscillator pool represented by the dot product of α^0^_*i, j*_ and *p_i, j_* (Equation 19).

(19) $$Q_{\text{learned},j}(T)=\sum_{i=0}^{N}\alpha_{i,j}^{0}p_{i,j}$$

Intra-pool phase relationship (i.e., within hip, within each knee) is maintained via the internal variable ψ_*i, j*_ (Equation 20) where τ forms the weight factor to maintain the phase relationship among the oscillators (Equation 14) with respect to the 0th oscillator within the pool (for a single “*j*” under consideration). This indicates that each oscillator within that pool receives a scaled phase input ψ_*i, j*_ from its respective reference oscillator ψ_0,*j*_

(20) $$\psi_{i,j}=\sin\text{​}\left(\frac{\omega_{i,j}}{\omega_{0,j}}\theta_{0,j}^{IP}-\theta_{i,j}^{IP}-\psi_{i,j}\right)$$

where the instantaneous phase of an oscillator, θ^IP^_*i, j*_ within a pool is

(21) $$\theta_{i,j}^{IP}=\text{sgn}(p_{i,j})\cos^{-1}\text{​}(-\frac{q_{i,j}}{z_{i,j}})$$

In addition to a local phase variable ψ_*i, j*_ (Equation 20), which does not consider the phase maintenance across different pools of oscillators (*j* = 1: s), a global/inter-pool phase relationship (between the hip and two knees) is introduced via a new state variable ψ^*G*^_0,*j*_, whose dynamics are governed by the following equations (Equations 22, 23). The Equation 22 is similar to Equation 14 with two changes that includes the variable *p*_0,*j*_, controlling the dynamics of only the 0th oscillator of each pool and the addition of the global phase variable ψ^*G*^_0,*j*_(Equation 23). These equations represent phase maintenance by a scaled phase input (ψ^*G*^_0,*j*_) across the pools of oscillators. In our case the global phase is maintained with respect to one of the hip oscillators (the 0th oscillator in the hip pool, i.e., ψ^*G*^_0,1_) as the reference oscillator. The block diagram for training the CPG network is given by Figure 6, and the Table 1 denotes the values of various parameters used in the CPG model.

(22) $$p_{0,j}=(\mu -z^{2})p_{0,j}-\omega_{0,j}q_{0,j}+\tau \sin(\theta_{0,j}^{IP}-\psi_{0,j}^{G})$$

(23) $$\psi_{0,j}^{G}=\sin(\theta_{0,j-1}^{IP}-\theta_{0,j}^{IP}-\psi_{0,j}^{G})$$

**Table 1:** **List of parameter values for simulating the network of adaptive hopf oscillators (CPG model)**.

**Parameters**	**Hip (*h*)**	**Knees (*k*1 and *k*2)**
ξ	8	12
μ	1	1
ϵ	0.9	0.3
τ	2	1

The GEN equation yields velocity components *v_x_* and *v_y_*, providing information on the magnitude and the direction of the agent's movements. Since our aim is to model the aspect of stride length, the magnitude of velocity obtained from the BG module is used to control the α^0^_*i, j*_ of the oscillators through a proportionality gain (*k*) (Equation 24). This provides a proxy for the magnitude of velocity in terms of the joint angles. Since the joint angular velocity can be varied in terms of their amplitude (by changing α^0^_*i, j*_), it is very convenient to translate an indirect measure of velocity obtained from the GEN module to a realistic motion of joints.

(24) $$k(t)=A_{k}\tanh(c_{k}\sqrt{v_{x}^{2}+v_{y}^{2}})$$

where *k*(*t*) is the proportionality gain variable. *A_k_* is the amplitude factor for the gain and *c_k_* is the sensitivity/slope factor which are set at 3 and 1 respectively in all conditions. The α^0^_*i, j*_ is modulated by *k*(*t*) as in Equation 25

(25) $$\alpha_{i,j}^{f}(t)=\alpha_{i,j}^{0}k(t)$$

**Figure 7:**
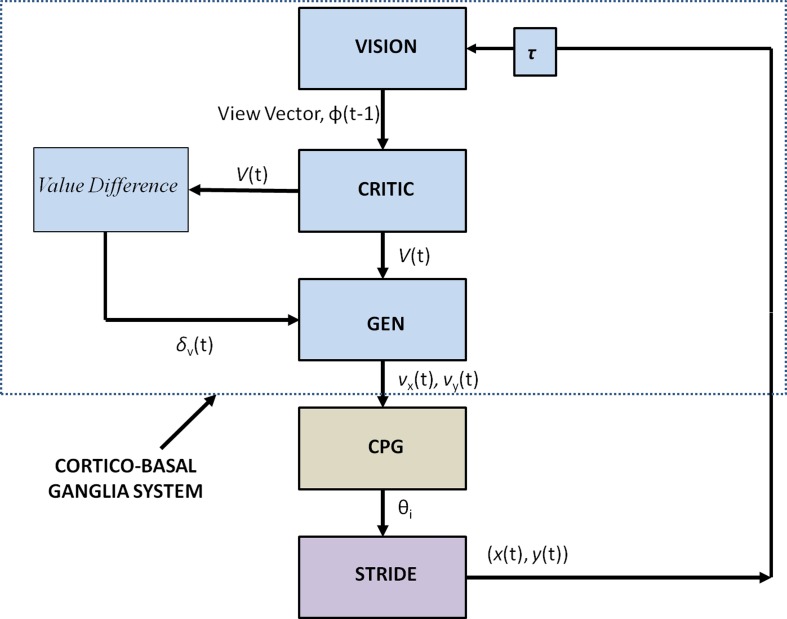
**Obtaining stride from the combined BG and the CPG network after training**.

Here α^*f*^_*i, j*_ reflects the changes in α^0^_*i, j*_ on being modulated by a factor of *k*(*t*) in each step of a trial to the doorway. Now α^*f*^_*i, j*_ takes up the role of α^0^_*i, j*_ (as seen in Equation 19) and has an effect on the output of the CPG network especially on dictating the amplitudes of the hip and knee angles. On obtaining larger/smaller values of velocity (*v_x_* and *v_y_*) from the GEN module the gain variable *k*(*t*) is varied i.e., either increased/decreased which in turn increases/decreases the amplitudes of the hip and knee angles by modulating α^0^_*i, j*_(Equation 25). Therefore, increased amplitude of velocity obtained from the BG results in increased stride lengths from the oscillator network and vice-versa. The stride module then calculates the stride/step length using θ_*h*_, θ_*k*1_ and θ_*k*2_, which is the actual displacement used to translate the agent's position in space. The stride length obtained as a function of the joint angles is described in the section below. The connectivity between the BG network and the CPGs for training is given by Figure 2. Once the CPGs are trained, the gait execution is modeled as in Figure 7.

### The locomotor apparatus: stride

As depicted in Figure 2 the stride module uses the angles θ_*i*_ (especially θ_*h*_) from the CPG network to determine the stride length. Stride length in a gait cycle is defined as the distance between the heel strike of one leg to the heel strike of the same leg and thus covers two steps. The hip angle θ_*h*_ as seen in Figure 5B has three peaks. Since θ_*h*_ is the angle between the two hips and knee angles are almost 0 at the extremes (Figure 5B) each peak in the hip angle represents a *Step*. Considering the first peak as the heel strike of one the legs, the next two peaks would be the next two steps or a *Stride* (Figure 5B). The thigh length, *l*_1_ is taken as 0.5 m and the shank length, *l*_2_ as 0.6 m (Figure 5A) and is adapted from Taga's biped model (Taga et al., 1991). The stride length (SL) is calculated as in Equation 26.

(26) $$L_{STR}=2(l_{1}+l_{2})\sin(\theta_{h\_ext2}/2)+2(l_{1}+l_{2})\sin(\theta_{h\_ext3}/2)$$

In order to simulate the *step lengths*, only a single peak (θ_*h_ext*2_) is considered; hence *L*_STR_will possess only the first term. As the α^0^_*i*_s are modulated, the amplitude of θ_*h*_ varies giving rise to different *stride / step lengths*. The stride / step length now supplies the displacement information to the agent. The information for direction is obtained from the unit vectors of *v_x_* and *v_y_* (${\hat{\nu }}_{x}$ and ${\hat{\nu }}_{y}$) of GEN module, respectively. The stride length and the direction are combined to calculate the agent's next position as in Equation 27.

(27) $$\begin{array}{l} \Delta x=L_{\text{STR}}{}^{*}{\hat{v}}_{x}\\ \Delta y=L_{\text{STR}}{}^{*}{\hat{v}}_{y} \end{array}$$

The change in position (performed by Equation 27) would then trigger the VISION module to compute the new view vector, thus forming a loop. The trained Cortico-Basal ganglia, CPG module along with the locomotor apparatus is then used for testing the agent's performance as shown in Figure 7.

## Results

We simulate the results of two experimental conditions that study gait patterns of PD patients as a they walk towards a doorway (Almeida and Lebold, 2010; Cowie et al., 2010). In both studies, PD patients were asked to walk through doorways of different sizes (wide, medium and narrow), with the idea of understanding the changes in gait velocity and the conditions that trigger FOG. The Cowie et al. (2010) study shows significant differences in the gait velocity and stride length, for healthy controls, PD ON and PD OFF freezers. The velocity and stride lengths were significantly different among the three subject groups. In this study, the controls produce higher velocities of gait and higher stride lengths than PD freezers, under all door conditions. PD ON subjects show lesser velocities and stride lengths compared to controls but higher than PD OFF, who show the lowest velocities. The PD subjects (both ON and OFF) also produce significant dips in their gait velocity especially near the doorway, showing signs of freezing (Figures 9A, 10A). The Almeida and Lebold (2010) study takes into account the gait patterns specifically of PD freezers and PD non-freezers. The differences among the three groups of subjects—controls, PD freezers and non-freezers—are evident from their step length profiles. PD freezers produce significantly low step lengths compared to controls and PD non-freezers. The trend is exaggerated as the doorway width decreases with the narrow doorway producing the least step length (Figure 12A). They also show increased variability among PD freezers in comparison to controls and non-freezers (Figure 13A). The experimental paradigm is quite similar in both the above studies (Almeida and Lebold, 2010; Cowie et al., 2010).

### Simulating the environment

We start with a description of the doorway and the reward schedule used in the ENVIRONMENT module of our model. The agent's state and action representation is in the form of view vector and the velocity vector respectively. The position vector limits are: [-2, 2] for *x*-position across the breadth of the track, and [0, 10] for *y*-position along the length of the track (Figure 3). At the start, the agent is always positioned at *y* = 0.1 for a random *x*, and is directly oriented toward the door, whose center is located at (*x, y*) = (0, 10) (Figure 3). The view vector ϕ(*t*) corresponding to any given position and orientation is given by eqn. 5, and the velocity is the action selected by following policy GEN (Equation 12). The agent is presented with three door conditions (wide, medium, and narrow).

In the Cowie et al. (2010) study, the door sizes are scaled to the participant's shoulders (100% shoulder width—narrow door; 125% shoulder width—medium door; 150% shoulder width—wide door), while the Almeida and Lebold study uses doors of fixed size (wide door—1.8 m; normal door—0.9 m; narrow door—0.675 m). In our model, the agent has a circular body of diameter 1 m and the door sizes (*d_length_*) are 3 m for “wide,” 2.5 m for “medium/normal” and 2 m for “narrow” cases. The agent must control its movements through a distance of 10 m before it encounters the door. The rewards/punishments are as follows: *r* = 5 at the door for successful passage, and *r* = −1 for collision with the sides of the door and the boundaries of the track; *r* = 0 elsewhere.

### Simulating the GEN

In the BG model, GEN parameters (*A*'s and λ 's: *A*_G_, *A*_N_,*A*_E_,λ_G_,λ_N_) of Equation 12 are computed for all the doorway cases (narrow, medium and “wide”) and medicated conditions (ON/OFF). For all the doorways, once the above parameters are first optimized for controls, they are then directly used for simulating the PD condition. The optimization is done such that the simulation results fall within the error of the experimental results. The cost function chosen for optimization considers two elements: (a) the magnitude of stride / step length for each doorway condition and (b) the stride / step length gradient between each doorway in any medication condition, the details of which are explained in Appendix A. The distinction between conditions of PD freezers (ON and OFF) and non-freezers among the two experiments is explained as follows.

#### In Cowie et al. (2010) study

Once the set for controls (*A*_G_, *A*_N_, *A*_E_, λ _G_, λ_N_, γ and σ) is optimized, the set for the PD freezers is obtained as follows. The parameters δ_lim_ (a status of limited dopamine availability), δ_med_ = 0 are treated specially for PD OFF case. Since δ_lim_ controls the clamping of δ (Equation 9), a step that represents dopamine deficiency under PD conditions, we search for the optimal δ_lim_(Equation 28) to describe PD OFF gait results. Furthermore, in PD OFF condition, we set δ_med_ = 0 denoting absence of medication. Additionally γ (discount factor) and σ (exploration parameter) are also trained in PD OFF condition. In summary, the parameters that are trained in PD OFF condition are δ_lim_, γ, and σ. The parameter δ_med_ is simply set to 0. All these parameters (*A*'s and λ 's from the control set, δ_lim_, γ) are carried over to PD ON from PD OFF case, except σ and δ_med_(Equation 29) which are trained. The optimized parameter values are as in Table 2.

**Table 2:** **Parameter values for different condition settings for δ _lim_, γ, σ, and δ_med_ for the Cowie et al. (2010) study**.

**Parameters**	**Controls**	**Freezers**	
		**PD OFF**	**PD ON**
δ_lim_	–	−0.1	−0.1
γ	0.8	0.1	0.1
σ	0.3	0.01	0.15
δ_med_	0	0	0.12

#### In Almeida and Lebold (2010) study

The controls set (*A*_G_, *A*_N_, *A*_E_, λ_G_, λ_N_, γ, and σ) is first optimized as in the Cowie et al. case. Further as the experimental results for both freezers and non-freezers are in PD ON condition, δ_lim_, δ_med_, γ and σ are optimized for PD freezers. For the PD non-freezers, all the PD freezers parameters are carried over except for γ and σ that are also optimized to match the experimental results. The optimized parameter values are as in Table 3.

**Table 3:** **Parameter values for different condition settings for δ_lim_, γ, σ, and δ_med_ for the Almeida and Lebold (2010) study**.

**Parameters**	**Controls**	**PD ON**	
		**Non-freezers**	**Freezers**
δ_lim_	–	−0.1	−0.1
γ	0.85	0.8	0.75
σ	0.23	0.22	0.02
δ_med_	0	0.12	0.12

The effect of adjusting parameters such as γ and σ in addition to δ (δ _lim_, δ_med_) for simulating PD freezers (ON / OFF as in Cowie et al., 2010) and non-freezers (as in Almeida and Lebold, 2010) compared to controls is apart from conventional modeling of PD condition where just the dopamine analogue δ in particular δ_lim_ and δ_med_ is varied. The motivation behind such a strategy is explained in Rationale Behind Optimization Strategy and Model Behavior Section.

The parameters including *A_G_*, *A_N_*, and *A_E_* are optimized to 2.5, 1 and 1 respectively; and the sensitivity to Go (λ_G_) and NoGo (λ_N_) is fixed at 1 and -1, respectively for the controls, PD freezers / non-freezers irrespective of the door-widths *d*_length_ simulated. Since PD is a dopamine-deficient condition, PD OFF conditions are simulated in the model by clamping δ (Equation 9) to a low value “δ_lim_” (Equation 28). To the clamped δ, a medication factor δ_med_ is added to simulate PD ON conditions (Equation 29). A similar modeling approach to PD conditions was adopted earlier in (Magdoom et al., 2011). Conceptually, if the range of δ values for controls is represented as [*a b*], then PD OFF adopts a range of [*a*δ_lim_] where both *a*, δ_lim_ < *b* and PD ON takes up the range of [*a* + δ_med_, δ_lim_ + δ_med_] where δ_lim_ + δ_med_ < *b*. In the simulations we set *a* and *b* as −1 and +1, respectively. Tables 2, 3 show the parameter values for different condition settings.

(28) $$\text{PDOFF}:\begin{array}{l} \text{If }\delta >\delta_{\lim}\\ \;\delta =\delta_{\lim} \end{array}$$

(29) $$\text{PDON}:\begin{array}{l} \text{If }\delta >\delta_{\text{lim}}\\ \;\delta =\delta_{\text{lim}}+\delta_{\text{med}}\\ \text{else}\\ \;\delta \text{=}\delta \text{+}\delta_{\text{med}} \end{array}$$

### Simulating the velocity profiles, stride / step lengths

The Cowie et al. (2010) study suggests that there is no significant change in cadence (steps/s) of the subjects involved in the study. Therefore, frequency of the hopf oscillators is fixed such that the output rhythm produces 2 steps/s or 1 stride. Moreover in order to prevent the agent from making undesirable backward movements away from the door, stride length/ step length is equated to a small constant value whenever the velocity (*v_y_*) generated from GEN is negative. In our simulation, this constant value is taken to be 0.0001. (Note that in the other case, the velocities *v_x_* and *v_y_* from GEN are translated into the corresponding stride / step length by using the CPGs of section The CPG Module). The model (cortico-BG system) simulation is discrete in time (*t*) i.e., each iteration is considered as execution of a single stride and a single update of the velocity of the agent.

During training, the agent repeatedly walks along a track to the doorway of specific size for 100 passes, and the value function is built up by training the value weights, *W* (Equation 8). In testing conditions, the pre-trained weights of the value function are used and the agent is run for another 100 passes to obtain a velocity profile along the track.

Since the model does not provide velocities at every point in space, linear interpolation is conducted to fill in the gaps of *v_y_*, which is averaged across the 100 passes to construct the velocity profile. Here, if *v_a_* and *v_b_* represent velocities at two discrete points *X_a_* and *X_b_*, then the velocities for intervals in between *X_a_* and *X_b_* is given by Equation 30.

(30) $$v_{\text{res}}=v_{a}+(v_{b}-v_{a})\frac{X_{\text{res}}-X_{a}}{X_{b}-X_{a}}$$

The following results are averaged over a length of the track starting from 2 m before the door till the doorway (in Y axis), for 50 such velocity profiles. In order to maintain regional consistency with each door size, the positions of the agent taken into account for averaging the velocities are (1) the position of the door itself and, (2) half of the door width [−2*d*_pos_,2*d_pos_*] on either side of the door along the width of the track.

### Rationale behind optimization strategy and model behavior

**Figure 8:**
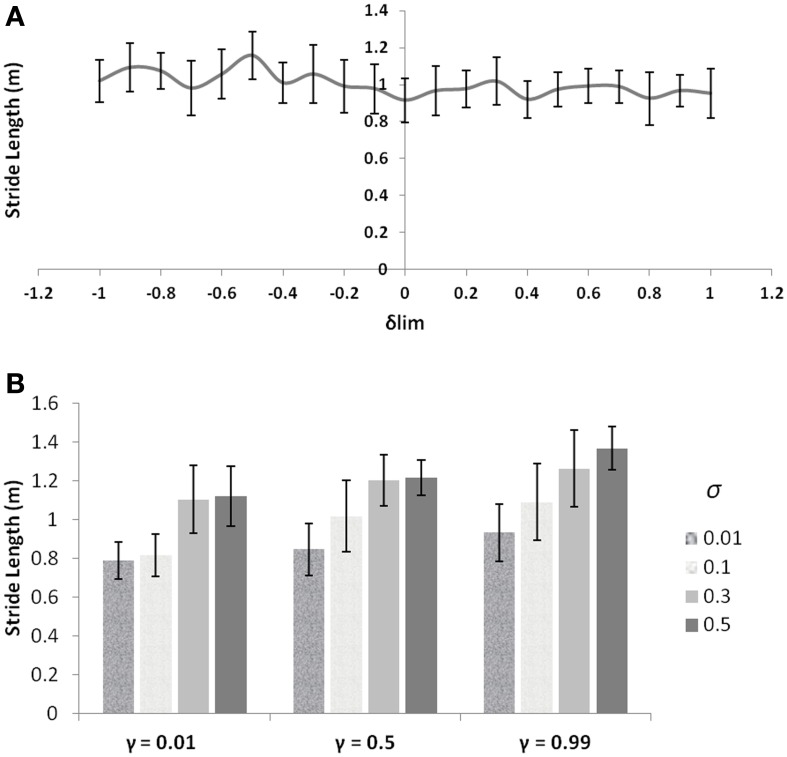
**(A)** Effect of δ_lim_ on stride lengths (simulations are run for γ = 0.8 and σ = 0.3) with other GEN parameters the same as that of the controls; **(B)** Effect of different levels of γ and σ on stride length (unclamped δ).

PD is a condition marked by decreased dopamine levels in the BG, and hence the simulations of the same from the controls are first directed toward understanding the role of the parameter δ_lim_. The dopamine analogue δ_lim_ is varied between [−1, 1], where −1 represents highly depleted conditions and +1 is the unclamped control conditions, and the stride length is determined at each level of δ_lim_ as the model output. The simulations are carried out in a freeze-stimulating narrow door case (following simulation criteria used for the Cowie et al. study, see Table 2) with all other parameters kept constant at control levels. The model shows no significant differences in the stride lengths on varying the parameter δ_lim_as seen in Figure 8A. Incidentally the Cowie et al. study makes an interesting observation between the velocity profiles of PD OFF and PD ON freezing subjects. The presence of the medication is not able to affect the stride trends for different doorways seen in both ON and OFF states (i.e., although PD ON subjects have increased strides to PD OFF, both class of subjects show sensitivity to doorway size), suggesting the involvement of factors other than dopamine in freezing events (Figure 10A).

These observations then forced us to investigate other parameters which could bring about such a behavior trend seen in the freezers. The discount factor γ and the exploration parameter σ are good modulatory candidates to explore apart from dopamine, owing to the fact that they are related to the neural correlates (Doya, 2002; Tanaka et al., 2007)—serotonin and norepinephrine respectively and also that their levels have been shown to be altered in PD and in medication conditions (Chalmers et al., 1971; Fahn et al., 1971). Varying the values of γ and σ, individually starts to produce changes in the stride lengths as seen in Figure 8B. These simulations (also following the simulation criteria used for Cowie et al. study, see Table 2) are carried out at unclamped or control level of δ_lim_ under a narrow doorway. The variation in stride length encourages the necessity in optimizing γ and σ in PD conditions to match the same trends seen in the experiments.

**Figure 9:**
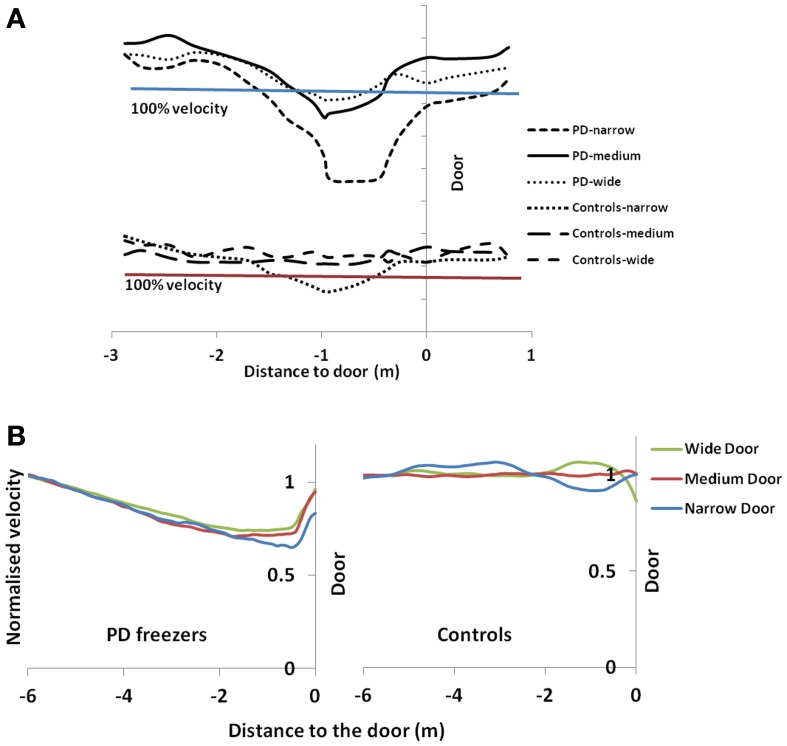
**Normalized velocity profile for controls and PD freezers in (A)** Experiment (Cowie et al., 2010) and **(B)** simulation under different doorway conditions. 100% velocity in the experimental results represents the velocity profile under a no-door condition. In simulation results, the velocity profiles are normalized by an average velocity far before (5–6 m) from the doorway.

**Figure 10:**
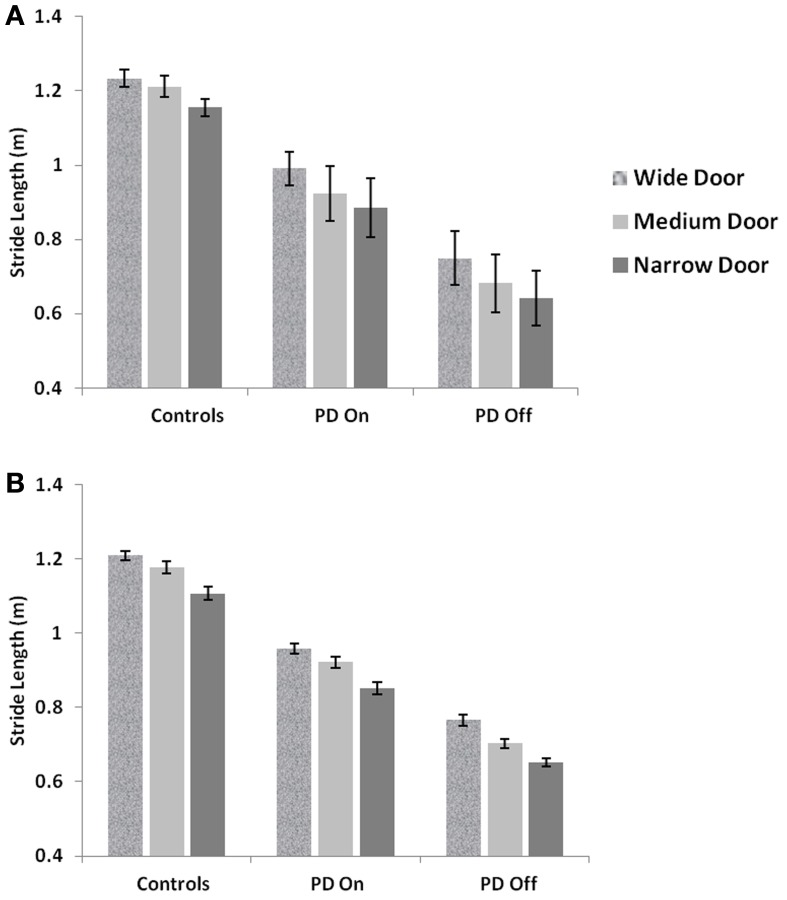
**Mean Stride lengths and Standard Errors for Controls, PD On and PD Off under different doorway conditions in (A) experiments (Cowie et al., 2010) and (B) simulations, reported with *p* < 0.005, *N* = 50**.

**Figure 11:**
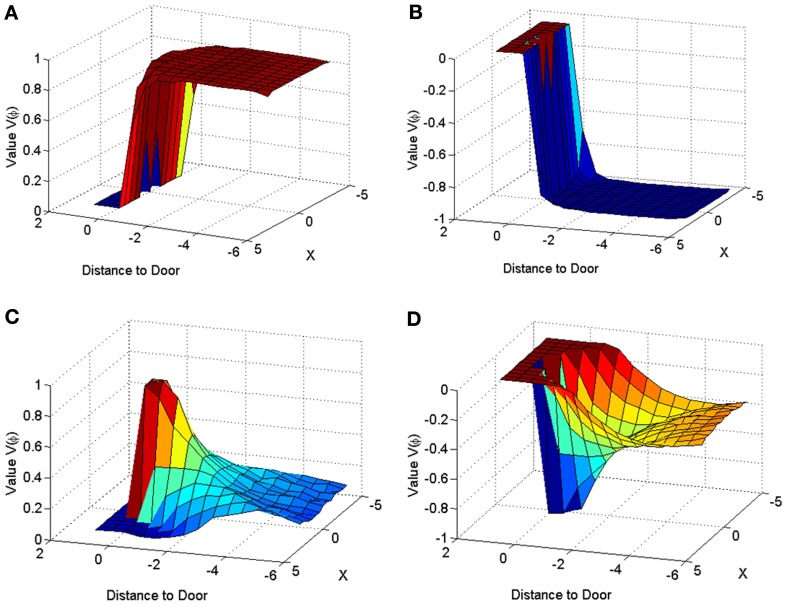
**Value function represented across space for a narrow door (*d*_length_ = 2) in Controls (A,C) and PD Condition (B,D)**. The graphs for **(A)** and **(B)** are obtained by using only the orientation vector facing toward the door at all points in space, while that of **(C,D)** are obtained by averaging the values corresponding to all the possible orientations, at a point in space.

The velocity profile obtained from the model of Cowie et al. (2010) for controls and the PD condition is as shown in Figures 9A,B respectively. In controls there seems to be a reduction in velocity on approaching the doorway which is exaggerated in PD conditions. The velocity near the doorway is normalized by the average velocity calculated far before the doorway (5–6 m) as seen in Figures 9A,B. Additionally simulations show a certain door-size dependent scaling of velocity in case of PD subjects. The simulated stride length profile for controls, PD ON and PD OFF under different doorway sizes is shown in Figure 10B, and that of the experiments (Cowie et al., 2010) in Figure 10A. The average stride length of controls is higher than that of the PD patients. In the model, we also found that PD ON case has higher mean velocities than PD OFF, in agreement with experimental data. Our simulation results also show that there is a significant difference in stride lengths (*p* < 0.005) between the wide/medium door and the narrow door conditions in both PD ON and PD OFF states (Figure 10). The shape of value function profile for both the controls and PD shows marked differences (Figure 11). Here, the value function for controls shows a positive gradient in the vicinity of the door suggesting the presence of a reward at the door. In case of PD patients, the value function is inverted and dips before the doorway, indicating low reward expectancy near the doorway. Since the GEN dynamics (Equation 12) depend on the gradient of value function (represented by δ_*V*_: Equation. 10), that negative gradient of value function may be a factor contributing to the velocity dip near the doorway.

**Figure 12:**
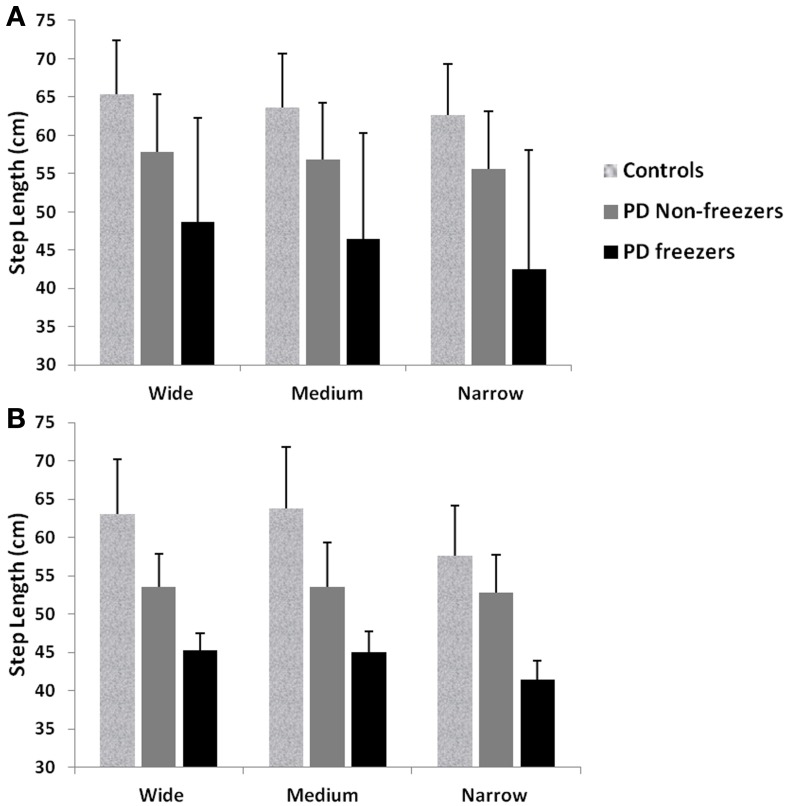
**Mean and Standard Deviation of Step length profiles for PD freezers and non-freezers under wide, medium and narrow door conditions in (A) experiments (Almeida and Lebold, 2010; Cowie et al., 2010), and (B) simulations**. PD freezers show significantly reduced step lengths compared to non-freezers (*p* < 0.05) and controls (*p* < 0.005) under all door conditions (*N* = 50).

**Figure 13:**
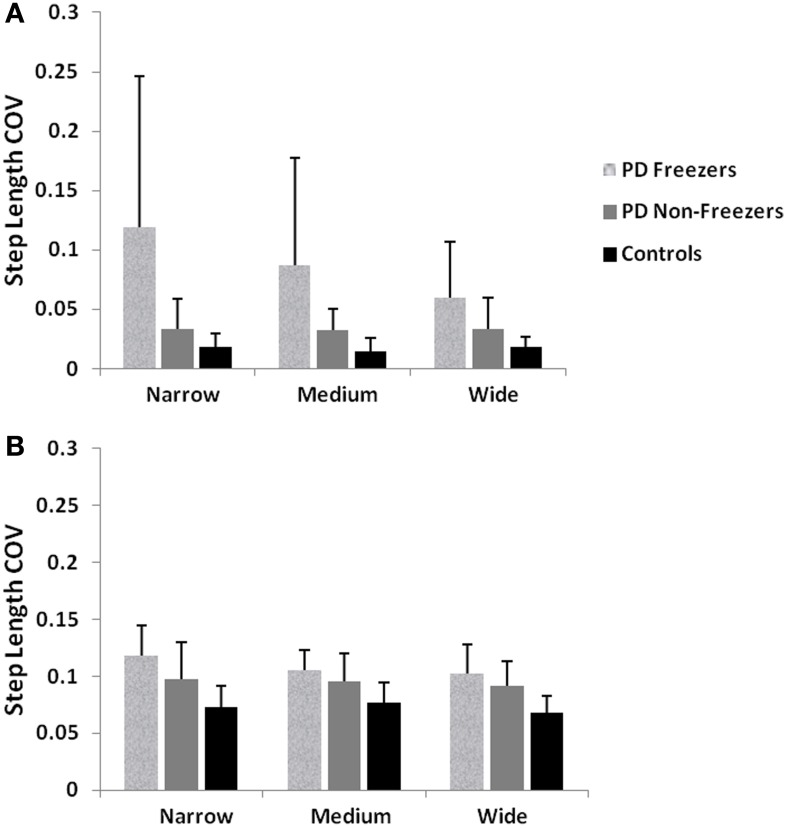
**(A)** Experimental Step length variability in controls, PD freezers and PD non-freezers (Almeida and Lebold, 2010), **(B)** Simulated Step length variability in controls, PD freezers and PD non-freezers. The significance between the conditions (Controls, PD freezers, PD non-freezers) and the cases (doorways: wide, medium, narrow) are reported with *p* < 0.05, *N* = 50.

Almeida and Lebold (2010) in their study show differences in gait patterns between PD ON—freezers and non-freezers. The experiments conducted in the ON condition report that the PD freezers group produces significantly reduced step lengths, compared to non-freezers and controls. This reduction in step lengths is further amplified in the case of reduced door sizes (*d*_length_). PD freezers also show changes in step length variability, a clear concomitant feature of freezing (Almeida et al., 2007). Our model captures this effect, and we present our results in terms of step length and step length coefficient of variation (CV). PD freezers show significantly reduced step lengths compared to non-freezers (*p* < 0.05) and controls (*p* < 0.005) under all door conditions (Figure 12). In order to capture the increased variability observed in PD freezers, the coefficient of variation (CV) in step length within a trial is determined (Figure 13B) throughout the corridor facing the doorway, and is averaged across trials (*N* = 50). Step length CV shows similar trends as seen in the original study (Figure 13A) where the PD freezers show significantly higher CV in comparison to controls and PD non-freezers in all the three door conditions. The step length CV reported in Almeida and Lebold (2010) is hypothesized to be a factor of unstable gait and a voluntary control over it.

A conclusion that the experiments lead to is that dopamine reduction, modeled here by clamping δ, alone cannot lead to FOG (Almeida and Lebold, 2010; Cowie et al., 2010). The simulations also reinforce the same conclusion. Therefore we studied the role of other model parameters including γ and σ in bringing about FOG (Almeida and Lebold, 2010; Cowie et al., 2010). This suggests the involvement of several factors for an event like FOG, and a single parameter (δ, γ, or σ) might not be sufficient to produce the observed effect of freezing. A plausible neurobiological interpretation of this modeling conclusion is presented in the following section.

## Discussion

In this study, we model gait changes and the occurrence of FOG in PD patients walking through doorways of different sizes. Our model reproduces the results of the studies of Cowie et al. (2010) and Almeida and Lebold (2010). The model shows significant decrease in the velocities (as a dip in velocity) and stride lengths for the PD (ON/OFF) compared to the Controls as seen in Cowie et al. (2010). The decrease in velocity observed in the controls and PD (ON/OFF) freezers, is also significant with changing door sizes i.e., the reduction in the door size increases the dip in velocity near the doorway. The step length profiles of Controls, PD freezers and PD non-freezers are also reproduced in concordance with the Almeida and Lebold (2010) results. We show that PD freezers produce significantly smaller steps than the controls and PD non-freezers in all the doorway conditions. Furthermore within the PD freezers (different doorway conditions), there exists a doorway effect with the narrowest door producing the least step length. In addition we replicate the trends observed that is the increased CV in step length found in PD freezers compared to non-freezers and controls.

FOG is a characteristic feature highlighting the cortical-BG loop influence on the spinal rhythms in the gait generation (Lewis and Barker, 2009; Naismith et al., 2010). Here, gait is a motor function that can be driven by spinal circuits and the error correcting systems like BG, with only a limited consciousness and voluntary control from the motor cortical areas (Takakusaki et al., 2008). Certain external conditions, for example confined spaces, might force a shift toward increased voluntary control (Maruyama and Yanagisawa, 2006) on gait. Furthermore the manifestation of FOG as start-hesitation, destination-hesitation and obstacle avoidance have been thought to be a result of impairment in willed / voluntary action (Maruyama and Yanagisawa, 2006). Lewis and Barker hypothesized that freezing might also result from the depletion in the available dopamine (δ), on induction of high cognitive loads (Lewis and Barker, 2009).

There are no existing computational models explaining the FOG in PD, to our knowledge. Our model captures this feat by carefully considering the impact of different levels of control on gait. The model consists of two stages of control: the cortico-BG and CPG on the locomotor apparatus. The cortico-BG module uses RL concepts for learning the environment in which the agent is placed (for navigating through doorway of variable widths). The BG dynamics are modeled through GEN that has been tested in many of our earlier studies (Sridharan et al., 2006; Magdoom et al., 2011; Kalva et al., 2012). This module outputs a higher level control parameter such as velocity of gait to be passed on to the next in control: the CPGs. The CPGs are modeled through dynamic adaptive hopf oscillators (Righetti and Ijspeert, 2006) representing the rhythmic spinal cord activity aiding the locomotion. Here the velocity obtained from the cortico-BG module is translated to the joint angle displacement during gait. This joint angle information is converted to the translatory motion in terms of stride / step lengths in the locomotor apparatus. This approach of modeling gives two major advantages: (1) It consolidates the essential functioning of the two stages of control in an abstract manner to explain the FOG, which a detailed model of only CPG driven biped model of gait (Taga et al., 1991; Mori et al., 2004) cannot reproduce. (2) It also explains the non-dopamine dependence on the FOG seen in the experiments modeled in this study (Almeida and Lebold, 2010; Cowie et al., 2010). The results point out the implications of the other parameters used in the study (γ and σ) for explaining the context dependent freezing phenomenon.

### Influence of δ, γ and σ parameters and their plausible correlates:

As discussed in the text above, δ is the dopamine functioning correlate depicting the temporal difference error in value function. Since dopamine deficiency is generally considered the crucial factor, the “star of the show” (Lewitt, 2012), responsible for PD related impairment, RL-based computational models of BG function typically propose TD error (a dopamine correlate) as the key variable that controls normal and pathological function. It has to be noted that the study by Cowie et al. (2010) made an interesting observation that the L-Dopa medication given to resurge the dopamine levels of PD freezers did not have a significant effect on the sensitivities to doorways. The same is captured by our model effectively, as seen in Figure 8A. The figure backs the non-dopamine dependence of FOG by showing no significant changes in stride length simulated for narrow doorway (width 2 m) under various clamped δ conditions simulated for control levels of γ and σ. It is also known that there are significant changes in other key neuromodulators like norepinephrine, serotonin and acetylcholine that is observed in PD, though these findings have not sufficiently influenced mainstream thinking about PD pathogenesis.

Norepinephrine is involved in important brain functions like wakefulness, vigilance and circadian rhythms (Aston-Jones et al., 1994; Yu and Dayan, 2005; Lewitt, 2012). Similar to loss of dopaminergic cells in SNc, there is marked loss of norepinephrine-releasing cells in Locus Coeruleus (LC) in PD (Cash et al., 1987; Del Tredici et al., 2002). Loss of norepinephrine is found to produce more pronounced motor impairment than destruction of dopamine fibers caused by MPTP (Rommelfanger and Weinshenker, 2007). Serotonin is known to be significantly involved in a wide spectrum of activities ranging from moods like anxiety, depression leading to major disorders such as bipolar disorder, major depression, schizophrenia, to reward- punishment sensitivity and their prediction in action selection (Lopez-Ibor, 1992; Vaswani et al., 2003; Boureau and Dayan, 2011; Rogers, 2011). There is evidence for altered serotonergic transmission and its involvement in motor impairment in PD (Fahn et al., 1971; Kish et al., 2008). It would be interesting to have a theory of BG function that combines the action of dopamine, norepinephrine and serotonin.

There was indeed an attempt to accommodate the function of all the four neuromodulators—dopamine, serotonin, norepinephrine and acetylcholine—in a unified theoretical framework based on RL (Doya, 2002). According to this view, dopamine represents TD error, norepinephrine represents exploration denoted by the temperature parameter, β, serotonin represents discount parameter, γ, and acetylchoine represents the learning rate, η. Specifically, within BG circuitry, it was suggested that GP is the substrate for exploration (Doya, 2002). GP is also known to have high levels of norepinephrine (Russell et al., 1992). From a purely dynamical point of view, chaotic dynamics of STN-GPe system qualifies to serve as a source of exploratory drive, an idea that has been investigated extensively using computational models (Sridharan et al., 2006; Ranganathan et al., 2012). In the present model, the exploration parameter, σ, denotes the extent of exploration, and therefore may be described as a neural correlate for norepinephrine in BG. Similarly serotonin has been linked to the discount factor, γ, or the time-scale of reward integration, with larger values of γ corresponding to higher levels of serotonin (Tanaka et al., 2007). Low levels of serotonin were associated with impulsivity, a behavior that may be thought to be a result of short-term reward seeking (Rogers, 2011). Based on the arguments just described, we adjust both γ and σ that represent serotonin and norepinephrine respectively, in addition to δ_lim_ and δ_med_ that are related to dopamine levels, in the present model to capture PD-related gait changes.

Therefore, in addition to incorporating PD-related changes in δ (δ_lim_ and δ_med_) corresponding to ON and OFF conditions respectively, we also explore the effect of the discount factor (γ) and exploration parameter (σ) on the velocity profile of the agent. These parameters have distinct roles in the model. By lowering σ it is possible to produce the velocity dip and stride length decrease as in Figures 8B, 9B. As a result PD freezers (ON / OFF) are modeled with lower σ compared to controls (See Table 2). The lower γ maintains the doorway effect between the controls and PD freezers and also emphasizes the fact that smaller values of γ fit PD velocity profiles better in the model, reflecting reduced serotonin levels in PD patients compared to controls (Figure 8B). Specifically, the PD ON conditions are modeled by increased σ compared to the PD OFF case and addition of δ_med_ in the model (described in Section Simulating the GEN). This assumption in modulating σ in addition to δ_med_ in PD ON implies that the medication factor δ_med_ increases the norepinephrine levels in the BG. There is evidence pointing to this claim and that the norepinephrine levels do increase on uptake of dopamine medication (Chalmers et al., 1971). L-Dopa treated rats have been found to have higher levels of norepinephrine mainly in the striatum, hypothalamus, brainstem and cerebellum (Romero et al., 1972). Taking into account these factors, the model incorporates the changes in σ which gives much better match to the experimental data than just altering δ_med_. This further led us to believe that even among PD subjects, the freezers could be hypothesized to have decreased serotonin and norepinephrine compared to non-freezers. Under the conditions of PD non-freezers, the γ and σ level increase in comparison to the PD freezers (Table 3). This results urge us to propose that γ and σ values may possibly reflect the importance of considering the other neuromodulators like serotonin and norepinephrine respectively, on context dependent FOG.

We conclude that the loss of dopaminergic cells alone cannot explain the FOG mechanism observed in PD patients. We predict that altered levels of serotonin and norepinephrine may contribute to freezing. Future work will be aimed at development of more detailed network model of BG and its role in gait control. The model will elucidate the contributions of dopamine, serotonin and norepinephrine to gait in normal and PD conditions.

### Conflict of interest statement

The authors declare that the research was conducted in the absence of any commercial or financial relationships that could be construed as a potential conflict of interest.

## Appendix

The Genetic Algorithm (Goldberg, 1989) option set for optimization is given in the following table. Optimization toolbox 6.0, Matlab R2011a, The Mathworks Inc. is used.

**Table A1:** **Option set for the GA tool**.

**Option**	**Value**									
Population size	20									
Crossover fraction	0.8									
Elite count	4									
Generation time	1000									
Function tolerance	1 e-6									
Bounds		*A_G_*	*A_N_*	*A_E_*	_,_λ_G_	λ_N_	γ	σ	δ_lim_	δ_med_
	Upper	0	0	0	0	0	0	0	−1	0
	Lower	10	10	10	1	−1	1	1	1	0.5
Cost function	(Expt measure—Sims measure)^2^									
	$$\begin{array}{l} C=\_0.5\sum_{i=1}^{3}{\left(ex_{i}-sims_{i}\right)}^{2}+0.5\{[(ex_{1}-ex_{2})+(ex_{2}-ex_{3})+(ex_{1}-ex_{3})]-\\ \left[(sims_{1}-sims_{2})+(sims_{2}-sims_{3})+(sims_{1}-sims_{3})]\right\} \end{array}$$									
	“*ex*” here refers to the experimental stride length values at each of the doorway (1—wide, 2—medium and 3—narrow) and “*sims*” is the model's ouput to a set of parameter values. The details of the parameters optimized at any given condition is described in section Result.									
